# Protective Effect of Iridoid Glycosides of the Leaves of Syringa oblata Lindl. on Dextran Sulfate Sodium-Induced Ulcerative Colitis by Inhibition of the TLR2/4/MyD88/NF-*κ*B Signaling Pathway

**DOI:** 10.1155/2020/7650123

**Published:** 2020-03-30

**Authors:** Yifang Zhang, Dandan Han, Shen Yu, Chiying An, Xin Liu, Haijing Zhong, Yuan Xu, Lianzhou Jiang, Zhongjiang Wang

**Affiliations:** ^1^Food Science College, Northeast Agricultural University, Harbin 150030, China; ^2^Department of Pharmaceutical Engineering, School of Chemical and Environmental Engineering, Key Laboratory of Green Chemical Engineering in Heilongjiang Province, Harbin University of Science and Technology, Harbin 150040, China; ^3^The First Affiliated Hospital of Harbin Medical University, Harbin 150001, China; ^4^Department of Pharmacology, School of Medicine, Yale University, New Haven, Connecticut 06520, USA

## Abstract

Iridoid glycoside (IG) is the major active fraction extracted from the leaves of *Syringa oblata Lindl*. In view of its antimicrobial and antidiarrheal potential, it could be beneficial for the treatment of ulcerative colitis (UC). In the present study, IG (20, 40, and 80 mg/kg) was administered orally for 14 days to dextran sulfate sodium- (DSS-) induced colitis rats. The anti-inflammatory effects of IG on DSS-induced UC were evaluated by comparing observations in DSS-induced colitis and drug-treated groups using disease activity index (DAI), macroscopic score, histological analysis, and apoptosis assay. To elucidate the antioxidant mechanisms of IG on NOX-dependent ROS production, the activities of 8-OHdG, NOX1, and NOX2 in DSS-induced colitis were determined. The levels of proinflammatory cytokines such as IL-2, IL-4, IL-5, IL-12p40, and IL-13 were detected. The inflammation-associated protein and mRNA expressions of TLR-2, TLR-4, MyD88, and NF-*κ*Bp65 were assessed by immunohistochemistry and real-time quantitative PCR, respectively. The results suggested that IG treatment significantly reduced DAI, macroscopic score, and histological damage compared to untreated animals (*p* < 0.01), whereas administration of IG remarkably attenuated the upregulation of 8-OHdG, NOX1, and NOX2 and the expression of proinflammatory cytokines such as IL-2, IL-4, IL-5, IL-12p40, and IL-13 in DSS-treated rats in a concentration-dependent manner. In addition, IG treatment could dose dependently suppress the protein and mRNA levels of TLR-2, TLR-4, MyD88, and NF-*κ*Bp65. The dose of IG that produced the most significant protective effect was 80 mg/kg. The above results demonstrate that IG exerts its inhibitory effect on cell apoptosis, oxidative stress, and proinflammatory cytokines in DSS-induced colitis through modulation of the TLR2/4/MyD88/NF-*κ*B signaling pathway.

## 1. Introduction

Inflammatory bowel disease (IBD) is becoming a global issue with accelerating incidence in newly industrialized countries during the past three decades [1]. Ulcerative colitis (UC), the major form of IBD, is a common inflammatory disease of the gastrointestinal tract, which is characterized by nonspecific, severe chronic relapsing course with clinically quiescent periods followed by bouts of severe intestinal inflammation, which are characterized by abdominal pain, diarrhea, weight loss, and fecal blood [2, 3]. Although the exact pathogenesis of UC has not been ascertained up to now, the abnormal mucosal immunity and colonic inflammation have been demonstrated to be major mechanisms involved in the pathophysiology of UC [3, 4]. The abnormal activity of the host immune system and inflammatory response are predominantly characterized by increased production of proinflammatory cytokines and the activation of reactive oxygen species (ROS) and reactive nitrogen species (RNS) [5, 6]. In this regard, modulation of both abnormal mucosal immunity and inflammatory response could be an important therapeutic modality for UC.

Increasing studies have suggested that the TLRs/MyD88/NF-*κ*B axis is a crucial inflammatory signaling pathway in the progression of UC and is involved in the process of immunization, colonic inflammation, oxidative stress, apoptosis, tumor occurrence, and other biological processes [7]. NF-*κ*B plays an important role in regulating the expression of inflammatory cytokines, chemokines, growth factors, COX-2, iNOS, and so on [3, 8]. Once activated, NF-*κ*B can translocate to the nucleus and tie in DNA binding sites, which induces the production of important immune mediators such as proinflammatory cytokines, ROS, and RNS [9, 10]. Oxidative stress in the intestinal tract is considered a major factor that contributes to the pathogenesis and progression of IBD, and NADPH oxidases (NOXs) are the main sources of ROS. The recent data suggest a positive correlation between upregulated NADPH oxidase (NOX) expression and gastrointestinal inflammation [11]. NOX possesses the special function of producing reactive oxygen. The previous studies also indicated that the epithelial NOX homologs, NOX1 and DUOX2, could produce a higher level of superoxide in the colon compared with phagocyte NOX2 [12]. Specially, the expression of NOX1 in human colonic epithelial cells is higher, and lymphocytes in lesions of CD and UC showed high levels of NOX1 expression [13]. In addition, oxidative stress contributes to the accumulation of oxidative damage products in the colon tissue such as 8-hydroxydeoxyguanosine (8-OHdG) [5]. Recent studies reveal that toll-like receptors (TLRs), such as TLR2, TLR4, and TLR6, are activated in the progression of UC [14]. TLR activation often stimulates the expression of proinflammatory cytokines, such as interleukin- (IL-) 2, IL-4, IL-5, IL-12p40, and IL-13 [5, 15]. Therefore, targeted inhibition of TLRs/MyD88/NF-*κ*B by removal or deactivation of inflammatory mediators and oxidative stress could be an important protective and therapeutic treatment for UC.

Syringa oblata Lindl., a green plant with the major fraction iridoid glycosides (IG), is widely used in China as a traditional Chinese medicine to treat intestinal inflammations [3]. As the most abundant active fraction extracted from the leaves of Syringa oblata Lindl., IG contain the high content of an active ingredient, syringopicroside [16]. In previous preclinical studies, we have evaluated their various potential applications such as antioxidative, anti-inflammatory, and immunomodulatory [3, 16]. However, the precise mechanism of anti-inflammatory effects by IG in dextran sulfate sodium- (DSS-) induced colitis is still limited, and few studies have described the antioxidative and antiapoptotic effects of IG in DSS-induced colitis.

Herein, our hypothesis of the immunoregulatory and anti-inflammatory effects of IG might associate with intervention on the TLRs/MyD88/NF-*κ*B signaling pathway. In the follow-up study, we investigated the protective effect of IG on DSS-induced UC in rat by assessing disease activity index (DAI), macroscopic scores, colon lengths, and histological changes. *In vivo* cytokine levels of IL-2, IL-4, IL-5, IL-12p40, and IL-13 were measured by an enzyme-linked immunosorbent assay (ELISA). The antioxidative role of IG was assessed by determining the activity of NOX1, NOX2, and 8-OHdG. Furthermore, the antiapoptosis role of IG was evaluated by TUNEL staining. The anti-inflammatory mechanisms of IG against the TLR2/4/MyD88/NF-*κ*B signaling pathway were further elucidated by detecting protein and mRNA expressions of TLR2, TLR4, MyD88, and NF-*κ*Bp65 using immunohistochemical staining and real-time quantitative polymerase chain reaction (PCR).

## 2. Material and Methods

### 2.1. Drugs and Reagents

The leaves of Syringa oblata Lindl. were collected in Heilongjiang Province in September 2019 (Voucher specimen no. 20190923) and identified by Professor Jianming Wang in Heilongjiang University of Chinese Medicine.

DSS (molecular weight 36~50 kDa) was provided by MP Biomedicals (Irvine, USA). Rabbit monoclonal antibody NF-*κ*Bp65 was obtained from Cell Signaling Technology Inc. (Beverly, MA, USA). Mouse monoclonal antibodies TLR2, TLR4, and MyD88 were purchased from Santa Cruz Biotechnologies (San Diego, USA). TdT-mediated dUTP nick end labeling (TUNEL) cell apoptosis detection kit was obtained from Roche Systems, Inc. (Basel, Switzerland). All cytokine enzyme-linked immunosorbent assay (ELISA) kits were supplied by R&D Systems (Minneapolis, USA). The primers for real-time PCR were synthesized by Invitrogen Biological Engineering Technology & Services Co., Ltd. (Beijing, China). All other chemicals were of reagent grade.

### 2.2. Animals

Sprague-Dawley (SD) rats (male, 200~220 g) were provided by the Center of Experimental Animals of Harbin Medical University (Harbin, China). The rats were housed at least 1 week to adapt to the new environment with a temperature of 22 ± 1°C and a relative humidity of 65% ± 5% under a 12 h light/dark cycle. Animal experiments were approved by the Institutional Animal Care and Use Committee of Northeast Agricultural University under the approved protocol number SRM-06.

### 2.3. Purification and Identification of Iridoid Glycosides

Iridoid glycoside (IG) was purified using D-141 macroporous adsorption resin column from the leaves of Syringa oblata Lindl. under the guidance of a previously established procedure [17]. Briefly, the dried leaves of Syringa oblata Lindl. (500 g) were pulverized to a powder and passed through a 20-mesh sieve. The powder was extracted for 120 min by refluxing with deionized water at 90°C and repeated two times. The filtered solution was concentrated under vacuum and precipitated by adding ethanol to a ratio of ethanol-water (70 : 30 (*v*/*v*)). The supernatant extracts were concentrated under reduced pressure and purified using a D-141 macroporous adsorption resin column. The product of IG fraction was obtained with a yield of 20.39% (101.95 g).

The HPLC analysis of IG fraction was performed by a previously described method [16]. ^1^H-NMR and ^13^C-NMR spectra of the most abundant active ingredient in IG fraction, syringopicroside, were recorded on a Bruker AVANCE 500 MHz NMR spectrometer (Switzerland) using tetramethylsilane (TMS) as an internal standard. The samples were dissolved in deuterated methanol (CD_3_OD) before NMR analysis. Fast atom bombardment-mass spectrometry (FAB-MS) was recorded on a Micromass Autospec Ultima ETOFJEOL mass spectrometer.

### 2.4. Induction of Colitis and Evaluation

Rats were randomly divided into five groups (*n* = 6 each group). Acute colitis was induced using a dose of 4% (*w*/*v*) DSS in drinking water for 7 days [3]. Rats in the normal group (I) received 0.9% saline solution only; by contrast, rats in the model group (II) received 4% DSS in drinking water. Following 7 days of DSS administration, rats in groups III~V received IG orally (20, 40, and 80 mg/kg, respectively) during DSS treatment once per day for 14 days. In the experimental period, weights of rats were recorded daily. DAI and macroscopic scores were evaluated based on the previously established scoring system [3, 16]. At the end of day 14, rats were sacrificed, and the colon was excised and measured. The colon tissues were fixed in 4% paraformaldehyde, then embedded in paraffin, and finally sectioned in 4 *μ*m sections. The samples were stained with hematoxylin and eosin (H&E) according to the standard procedures for histological evaluation [16].

### 2.5. Apoptosis Assay

The colonic cell apoptosis was assessed using the terminal deoxynucleotidyl transferase- (TdT-) mediated dUTP-biotin nick end labelling (TUNEL) kit. TUNEL-positive epithelial cells in colonic tissue were clearly identified as brown-stained nuclei, which suggested DNA fragmentation due to apoptosis. TUNEL-positive expression was detected via 1000 cells in random fields.

### 2.6. Evaluation of the Activities of 8-OHdG and NOX

Colon samples were homogenized in 10 volumes of 0.1 M Tris-HCl buffers (pH 7.4) using an electric homogenizer (IKA T10, Germany) in an ice bath. The homogenate was centrifuged at 10000 rpm at 4°C for 30 min and obtained the supernatant. The levels of 8-OHdG were determined using ELISA Kits (Nanjing Jiancheng Biological Engineering Institute, Jiangsu, China) following the manufacturer's protocol. The activities of NOX1 and NOX2 were detected as previously reported [18]. In brief, a 20 *μ*l supernatant of homogenized and centrifuged colon samples was added into a 96-well luminescence plate, then mixed with 80 *μ*l PBS and 6.25 *μ*l l M lucigenin. NADPH was added to start the reaction, and photoemission was determined by the absorbance at 340 nm, which is monitored every 30 s for 5 min.

### 2.7. Determination of Inflammatory Cytokine

The colon tissues were homogenized in ice-cold physiological saline at the final concentration of 10% (*w*/*v*). Cytokine levels of IL-2, IL-4, IL-5, IL-12p40, and IL-13 in colon tissue homogenates (1/5 dilution) were quantified using ELISA kits according to the manufacturer's instructions.

### 2.8. Immunohistochemical Staining

The protein expressions of TLR2, TLR4, MyD88, and NF-*κ*Bp65 were detected according to a method described previously [3]. Briefly, 4 *μ*m colon sections were first treated with 3% hydrogen peroxidase for 10 min to block endogenous peroxidase, then incubated with the polyclonal primary antibody of TLR2, TLR4, MyD88, and NF-*κ*Bp65 (diluted to 1 : 100) overnight at 4°C. The colon sections were then washed with phosphate-buffered saline (PBS) and incubated with polyclonal rabbit anti-mouse biotinylated secondary antibody (Dako, CA, USA). After that, colon sections were incubated with 3,3′-diaminobenzidine solution (Sigma-Aldrich, St. Louis, MO, USA) and then stained with hematoxylin. Finally, images were observed under an Olympus BH-2 microscope (Tokyo, Japan).

### 2.9. Real-Time PCR

Colon tissues were homogenized in a lysis buffer for RNA isolation. Total RNA isolation from colonic cells was performed using the TRIzol reagent (Invitrogen, Carlsbad CA, USA) according to the manufacturer's manual. RNA was transcribed into cDNA using the first-strand cDNA synthesis kit (Fermentas International Inc., Burlington, Canada) in accordance with the manufacturer's instructions. Primer sequences for real-time PCR analysis are shown in Table 1. mRNA expressions were normalized to GAPDH (18 S rRNA endogenous control) and calculated according to the 2^-*ΔΔ*Ct^ method (*n* = 6).

### 2.10. Statistics

All the results were presented as the mean ± standard deviation (mean ± SD). Statistical analysis was performed with SPSS 19.0 statistical software. Statistical analysis used a one-way ANOVA test. Differences with *p* < 0.05 and *p* < 0.01 were considered statistically significant.

## 3. Results

### 3.1. Identification and Quantification of Iridoid Glycosides

**Figure 1:**
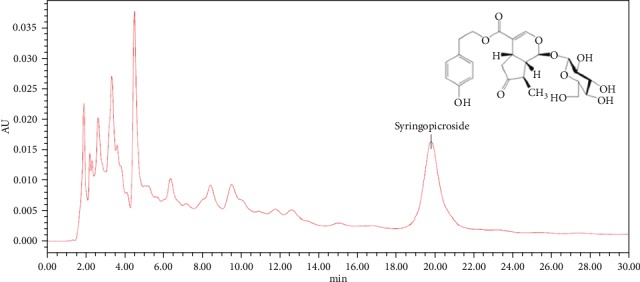
Chemical structure of syringopicroside, the major ingredient in iridoid glycoside (IG) fraction. HPLC chromatogram of IG fraction purified using D141 macroporous adsorption resin is measured at 221 nm.

HPLC analysis confirmed that the major ingredient in IG fraction is syringopicroside (Figure 1). The content of syringopicroside in the iridoid glycosides fraction reached 57.83%, which was 25-fold to that in the crude extracts. ^1^H-NMR parameters of syringopicroside were as follows: *δ* 7.00 (2H, d, *J* = 8.5 Hz, H-2^″^, H-6^″^), *δ* 6.68 (2H, dd, *J* = 8.5, 2 Hz, H-3^″^, H5^″^), *δ* 2.80 (2H, t, *J* = 6.5 Hz, H-*α*), *δ* 4.21 (2H, t, *J* = 6.5 Hz H-*β*), *δ* 5.57 (1H, d, *J* = 3 Hz, H-1), *δ* 7.40 (1H, d, *J* = 6.5 Hz, H-3), *δ* 2.86 (1H, dd, *J* = 12, 2 Hz, H-5), *δ* 2.51 (1H, dd, *J* = 19.2, 8 Hz, H-6*α*), *δ* 2.37 (1H, dd, *J* = 19.2, 8 Hz, H-6*β*), *δ* 2.06 (1H, dq, H-8), *δ* 2.28 (1H, ddd, *J* = 14, 7.2, 3.5, H-9), *δ* 4.64 (1H, d, *J* = 8 Hz, H-1), *δ* 3.62 (1H, dd, *J* = 14, 6.5 Hz, H-*α*), and *δ* 3.56 (1H, dd, *J* = 14, 6.5 Hz, H-6′*β*); ^13^C-NMR parameters of syringopicroside were as follows: *δ* 95.41 (C-1, -CH), *δ* 153.19 (C-3, -CH), *δ* 111.18 (C-4, -C), *δ* 28.22 (C-5, -CH), *δ* 43.5 (C-6, 43.5), *δ* 220.74 (C-7, -C), *δ* 46.62 (C-8, –CH), *δ* 44.46 (C-9, -CH), *δ* 13.67 (C-10,-CH_3_), *δ* 168.37 (C-11, -C), *δ* 100.19 (C-1′, -CH), *δ* 74.62 (C-2′, -CH), *δ* 77.93 (C-3′, -CH), *δ* 71.53 (C-4′, -CH), *δ* 78.36 (C-5′, -CH), *δ* 62.71 (C-6′, -CH_2_), *δ* 130.09 (C-1^″^, -C), *δ* 130.90 (C-2^″^, C-6^″^, -CH), *δ* 116.29 (C-3^″^, C-5^″^, -C), *δ* 157.02 (C-4^″^, -C), *δ* 66.29 (C-*α*, -CH_2_), and *δ* 35.26 (C-*β*, -CH_2_). The parameters of syringopicroside were analyzed by FAB-MS: m/e 495.3, 333.3, 245.2, 195.1, 185.1, and 121 (C_24_H_30_O_11_).

### 3.2. Iridoid Glycosides Ameliorate DSS-Induced Colitis

**Figure 2:**
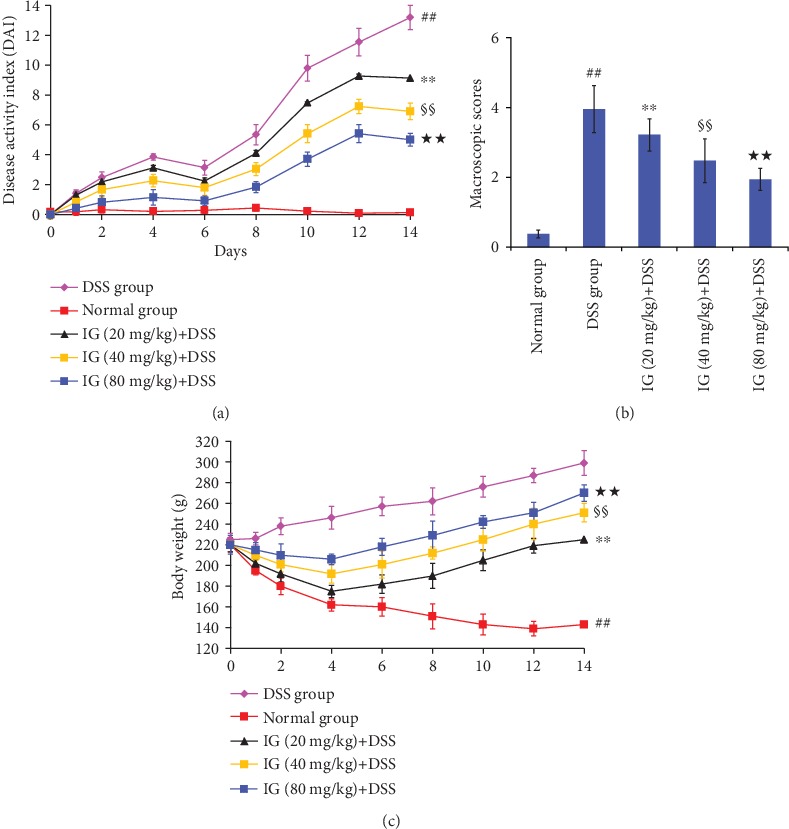
Iridoid glycosides attenuated changes in DAI score, macroscopic score, and body weight loss in DSS-induced colitis. Rats were treated orally at different doses of IG 20, 40, and 80 mg/kg once for 14 days after administration of DSS. The severity of colonic injury and the clinical evaluation were measured by (a) DAI score, (b) macroscopic score, and (c) body weight. IG administration dose dependently attenuated these pathological parameters. Data are presented as the mean ± SD (*n* = 6 per group). ^##^*p* < 0.01 vs. the normal control group, ^∗∗^*p* < 0.01 vs. the DSS-induced colitis group, ^§§^*p* < 0.01 vs. the IG (20 mg/kg)+DSS group, and ^★★^*p* < 0.01 vs. the IG (40 mg/kg)+DSS group.

**Figure 3:**
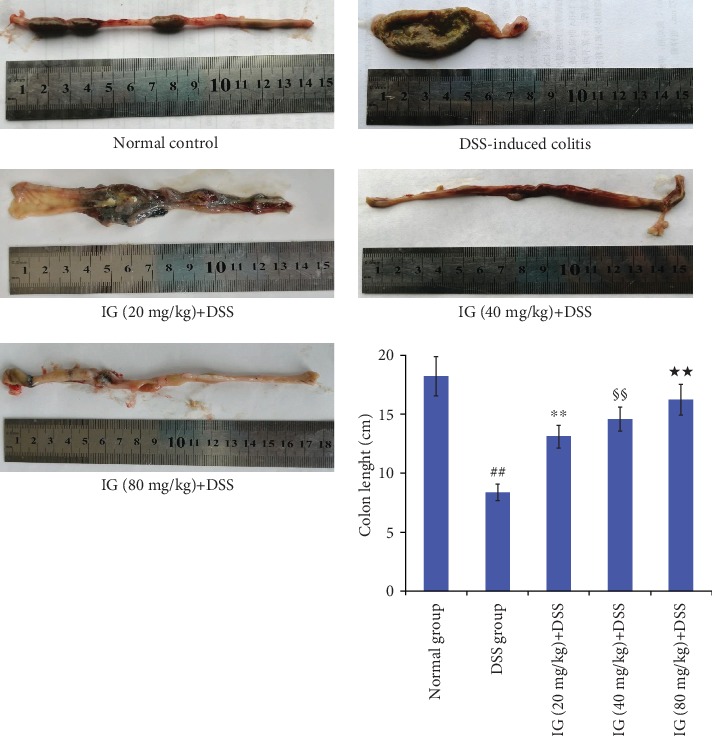
Iridoid glycosides attenuated colon length shortening in DSS-induced colitis. Rats were treated orally at different doses of IG 20, 40, and 80 mg/kg once for 14 days after administration of DSS. IG administration dose dependently attenuated the colon length shortening. Data are presented as the mean ± SD (*n* = 6 per group). ^##^*p* < 0.01 vs. the normal control group, ^∗∗^*p* < 0.01 vs. the DSS-induced colitis group, ^§§^*p* < 0.01 vs. the IG (20 mg/kg)+DSS group, and ^★★^*p* < 0.01 vs. the IG (40 mg/kg)+DSS group.

Severe DSS-induced colitis was observed and characterized by obvious hyperemia, edema, stool consistency, and ulceration. In this study, DAI and macroscopic scores in the DSS-induced colitis group were higher than those in the normal control group. However, IG treatment significantly attenuated DAI and macroscopic scores during experimental colitis in comparison with the DSS group (Figures 2(a) and 2(b)). The difference among the three dose groups of IG was statistically significant (*p* < 0.05). Moreover, DSS intake obviously induced colonic shortening and weight loss. The mean colon length (63 ± 2.9 mm) in the DSS-induced model group was lower than that in the normal group (92 ± 6.3 mm, *p* < 0.01). Animals show significant weight loss accompanied with obvious diarrhea in the DSS model group compared to the normal control group. The decrease in colon length and body weight after DSS administration was gradually reversed by IG in a dose-dependent manner (*p* < 0.01, Figures 2(c) and 3). As described above, treatment with IG dose dependently improved these pathological symptoms.

### 3.3. Iridoid Glycosides Suppressed Histopathological Damage and Apoptosis

**Figure 4:**
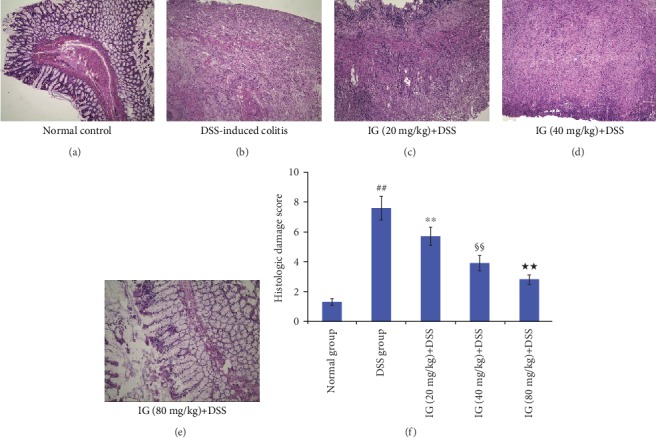
Iridoid glycosides alleviated DSS-induced histologic damage in a dose-dependent manner. The protective role of different doses of IG on DSS-induced histological damage was evaluated by H&E staining. Histopathological scores of each group were assessed. IG treatment significantly improved the histological damage in a dose-dependent manner. Colonic tissue sections were observed under a light microscope (100x). Data are presented as the mean ± SD (*n* = 6 per group). ^##^*p* < 0.01 vs. the normal control group, ^∗∗^*p* < 0.01 vs. the DSS-induced colitis group, ^§§^*p* < 0.01 vs. the IG (20 mg/kg)+DSS group, and ^★★^*p* < 0.01 vs. the IG (40 mg/kg)+DSS group.

H&E staining showed that DSS administration distorted glandular formation and led to the recruitment of inflammatory cells into the submucosal layer, leading to necrosis, hyperemia, and mucosal destruction (Figure 4(b)). On the other hand, the histologic damage score in the DSS-induced colitis group was higher than that in the control group (Figure 4(f)). IG treatment remarkably attenuated these pathologic changes with a lower histologic damage score in a dose-dependent manner (*p* < 0.01, Figures 4(c)–4(f)). The results are shown in Figure 4.

**Figure 5:**
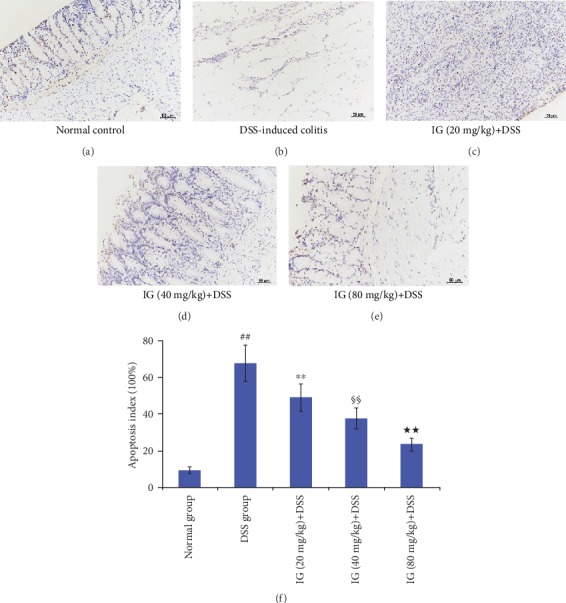
Iridoid glycosides attenuated apoptosis in DSS-induced colitis. The effect of different doses of IG on apoptosis in DSS-induced colitis was assessed by TUNEL staining. Apoptosis index scores of each group were evaluated. IG treatment significantly attenuated apoptosis in a dose-dependent manner. The scale bar is 50 *μ*m. Data are presented as the mean ± SD (*n* = 6 per group). ^##^*p* < 0.01 vs. the normal control group, ^∗∗^*p* < 0.01 vs. the DSS-induced colitis group, ^§§^*p* < 0.01 vs. the IG (20 mg/kg)+DSS group, and ^★★^*p* < 0.01 vs. the IG (40 mg/kg)+DSS group.

Additionally, TUNEL staining indicated that colon tissues exhibited a significant increase of brown apoptotic cells and intercellular apoptotic fragments after treatment with DSS (*p* < 0.01, Figure 5(b)), whereas few TUNEL staining-positive cells were observed in the normal group (Figure 5(a)). In contrast, a low-dose group of IG (20 mg/kg) reduced the number of apoptotic epithelial cells (Figure 5(c)). Administration of a middle dose of IG (40 mg/kg) showed a remarkable decrease of TUNEL-positive cells compared to the low-dose group (*p* < 0.01, Figure 5(d)). In particular, IG in a high dose of 80 mg/kg was the most effective in suppressing intestinal epithelial cell apoptosis (*p* < 0.01, Figure 5(e)). The results are shown in Figure 5.

### 3.4. Iridoid Glycosides Attenuated Oxidative Stress

**Figure 6:**
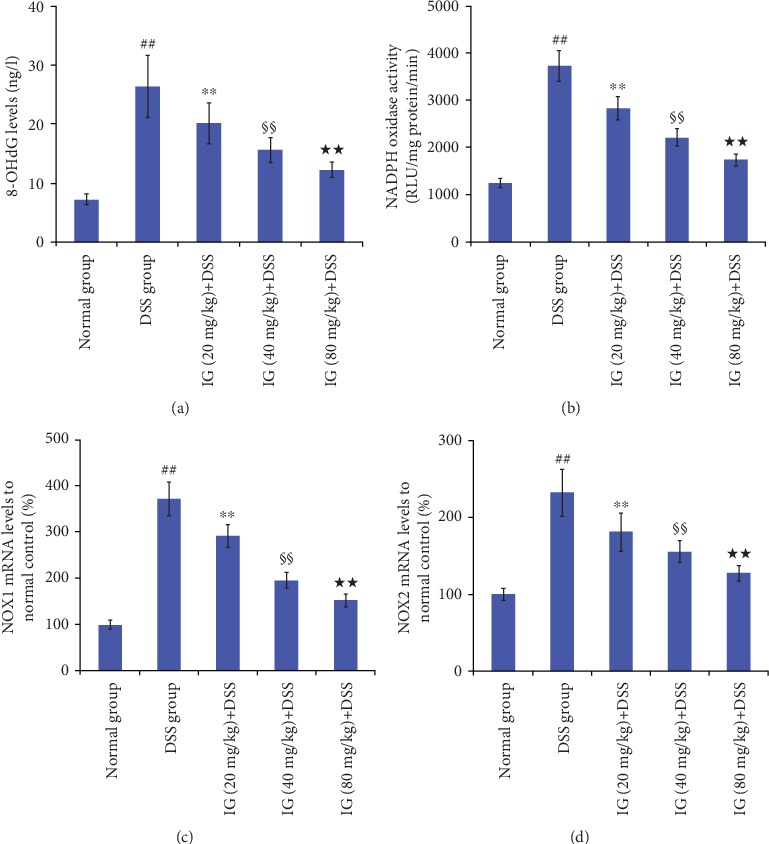
Iridoid glycosides attenuated the expression of 8-OHdG, NOX1, and NOX2, in a DDS-induced colitis model. (a) 8-OHdG expression in the colon tissues, (b) NOX activity in the colon tissues of the five experimental groups, (c) mRNA expressions of NOX1 in the colon tissues compared to the normal control group, and (d) mRNA expressions of NOX2 in the colon tissues compared to the normal control group. Data are presented as the mean ± SD (*n* = 6 per group). ^##^*p* < 0.01 vs. the normal control group, ^∗∗^*p* < 0.01 vs. the DSS-induced colitis group, ^§§^*p* < 0.01 vs. the IG (20 mg/kg)+DSS group, and ^★★^*p* < 0.01 vs. IG (40 mg/kg)+DSS group.

To evaluate the antioxidant effects of IG in a DSS-induced colitis model, we analyzed the levels of 8-OHdG, NOX1, and NOX2. In the DSS-induced colitis group, the expression levels of 8-OHdG, NOX1, and NOX2 in colon tissue were significantly higher than those in the normal control group. In addition, IG treatment could markedly suppress this colitis-induced increase in the levels of these three oxidative stress indicators (*p* < 0.01). The results suggest a negative correlation between the increased dose of IG and the expression of 8-OHdG and NADPH oxidase (NOX), as shown in Figures 6(a)–6(d).

### 3.5. Iridoid Glycosides Alleviate Inflammatory Cytokine

**Figure 7:**
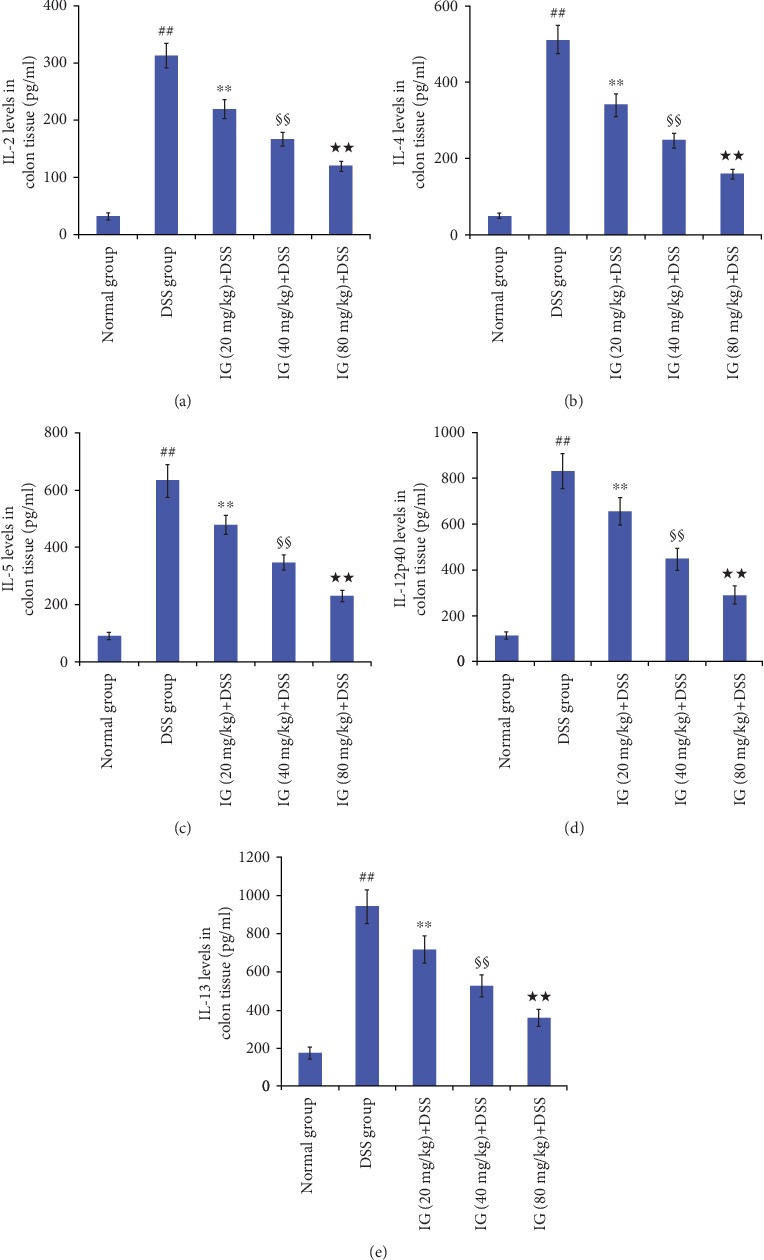
Iridoid glycosides reduced levels of DSS-induced inflammatory cytokines in colon tissue. Analysis of ELISA showed that the levels of IL-2, IL-4, IL-5, IL-12p40, and IL-13 were markedly decreased in the IG-treated group. Data are presented as the mean ± SD (*n* = 6 per group). ^##^*p* < 0.01 vs. the normal control group, ^∗∗^*p* < 0.01 vs. the DSS-induced colitis group, ^§§^*p* < 0.01 vs. the IG (20 mg/kg)+DSS group, and ^★★^*p* < 0.01 vs. the IG (40 mg/kg)+DSS group.

The levels of inflammatory cytokine in colon tissue were significantly upregulated in the DSS-induced colitis group compared to the normal control group (*p* < 0.01). In addition, IG treatment could remarkably reverse the levels of IL-2, IL-4, IL-5, IL-12p40, and IL-13 in a dose-dependent manner (*p* < 0.01). The maximum inhibition effect was observed with IG at a dose of 80 mg/kg, as shown in Figures 7(a)–7(e).

### 3.6. Iridoid Glycosides Inhibited DSS-Induced TLR2/4/MyD88/NF-*κ*B Signaling Pathway Activation

**Figure 8:**
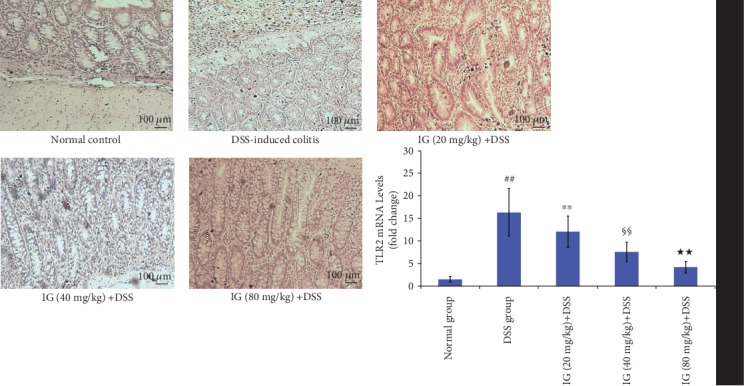
Iridoid glycosides reduced mRNA and protein expressions of TLR2 in DSS-induced colitis. The protein levels of TLR2 were analyzed by immunohistochemical staining. The relative mRNA expressions of TLR2 was normalized to GAPDH. Data are presented as the mean ± SD (*n* = 6 per group). ^##^*p* < 0.01 vs. the normal control group, ^∗∗^*p* < 0.01 vs. the DSS-induced colitis group, ^§§^*p* < 0.01 vs. the IG (20 mg/kg)+DSS group, and ^★★^*p* < 0.01 vs. the IG (40 mg/kg)+DSS group.

**Figure 9:**
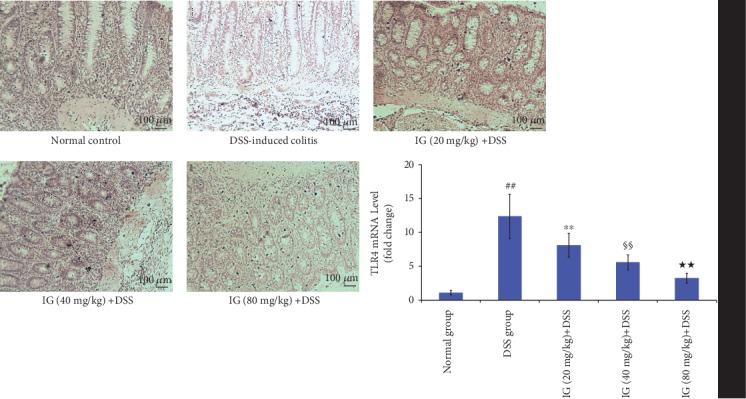
Iridoid glycosides reduced mRNA and protein expressions of TLR4 in DSS-induced colitis. The protein levels of TLR4 were analyzed by immunohistochemical staining. The relative mRNA expressions of TLR4 were normalized to GAPDH. Data are presented as the mean ± SD (*n* = 6 per group). ^##^*p* < 0.01 vs. the normal control group, ^∗∗^*p* < 0.01 vs. the DSS-induced colitis group, ^§§^*p* < 0.01 vs. the IG (20 mg/kg)+DSS group, and ^★★^*p* < 0.01 vs. the IG (40 mg/kg)+DSS group.

**Figure 10:**
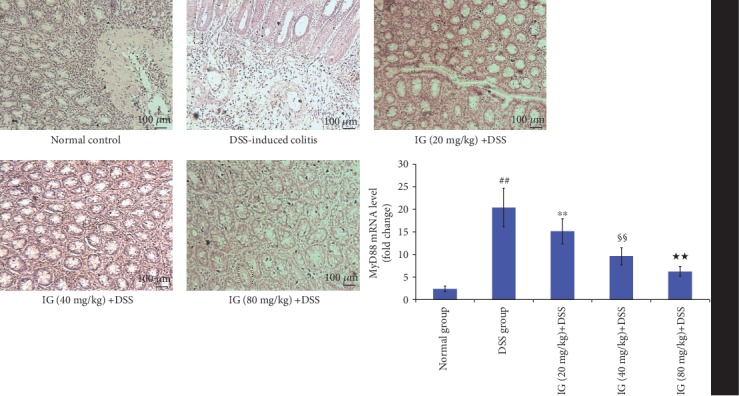
Iridoid glycosides reduced mRNA and protein expressions of MyD88 in DSS-induced colitis. The protein levels of MyD88 were analyzed by immunohistochemical staining. The relative mRNA expressions of MyD88 were normalized to GAPDH. Data are presented as the mean ± SD (*n* = 6 per group). ^##^*p* < 0.01 vs. the normal control group, ^∗∗^*p* < 0.01 vs. the DSS-induced colitis group, ^§§^*p* < 0.01 vs. the IG (20 mg/kg)+DSS group, and ^★★^*p* < 0.01 vs. the IG (40 mg/kg)+DSS group.

**Figure 11:**
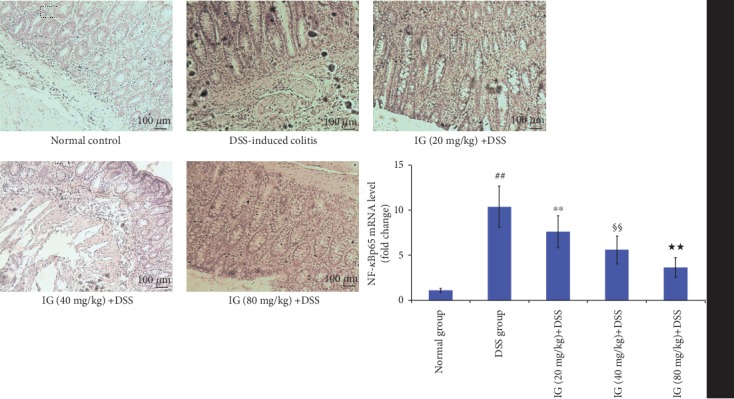
Iridoid glycosides reduced mRNA and protein expressions of NF-*κ*Bp65 in DSS-induced colitis. The protein levels of NF-*κ*Bp65 were analyzed by immunohistochemical staining. The relative mRNA expressions of NF-*κ*Bp65 were normalized to GAPDH. Data are presented as the mean ± SD (*n* = 6 per group). ^##^*p* < 0.01 vs. the normal control group, ^∗∗^*p* < 0.01 vs. the DSS-induced colitis group, ^§§^*p* < 0.01 vs. the IG (20 mg/kg)+DSS group, and ^★★^*p* < 0.01 vs. the IG (40 mg/kg)+DSS group.

The TLR2/4/MyD88/NF-*κ*B signaling pathway is an important signaling pathway in regulating inflammation. Its activation has been demonstrated to be related to the development of UC [14]. The activation of the TLR2/4/MyD88/NF-*κ*B signaling pathway enhances the production of various proinflammatory cytokines and the induction of oxidative stress and apoptosis [10, 14]. To explore the inhibitory mechanism of IG on oxidative stress and cytokines, the key protein expressions in the TLR2/4/MyD88/NF-*κ*B signaling pathway were detected using real-time PCR and immunohistochemical staining. The TLR2/4/MyD88/NF-*κ*B signaling pathway was significantly activated in the DSS-induced colitis group. The mRNA and protein levels of TLR2, TLR4, MyD88, and NF-*κ*Bp65 were all increased compared to the normal control group (*p* < 0.01). As expected, IG dose dependently suppressed the mRNA and protein levels of TLR2, TLR4, MyD88, and NF-*κ*Bp65 in comparison with the DSS group (*p* < 0.01). These data verified our hypothesis that IG might have an anti-inflammatory effect by blocking TLR2/4/MyD88/NF-*κ*B activation. The results are shown in Figures 8–11.

## 4. Discussion

DSS is a chemical colitogen with anticoagulant properties that induce damage in the epithelial monolayer lining in the intestine, leading to the dissemination of proinflammatory intestinal contents including ROS production, bloody diarrhea, weight loss, colon shortening, and mucosal ulceration [5, 19]. Hence, DSS-induced colitis models are commonly used to study for IBD. DAI, macroscopic score, colon length shortening, and body weight loss are the main parameters in the evaluation of the severity of UC. In the present study, DAI, macroscopic score, colon length shortening, and weight loss were significantly increased after DSS treatment. From histological analysis and TUNEL staining, we also found that DSS intake caused remarkably inflamed tissue and had marked necrosis, hyperemia, numerous granulocytes, and high levels of apoptosis. Our findings demonstrated that administration of IG attenuated DSS-induced colonic injury dose dependently as assessed by these pathological parameters and the histologic damage. In addition, we indicated that IG treatment markedly suppressed DSS-induced high levels of apoptosis in a dose-dependent manner.

Excessive reactive oxygen species (ROS) in the physiopathology of UC contributes to oxidative damage and inflammatory cascade, and NADPH oxidases (NOXs) are the main sources of ROS [6]. Increasing evidence shows that oxidative stress leads to the accumulation of 8-OHdG in colon tissue [5]. Our previous reports have demonstrated that IG treatment could lead to a significant reduction in oxidative stress levels by virtue of decreasing levels of malondialdehyde (MDA) and NO in a trinitrobenzene sulfonic acid-induced colitis rat model [16]. However, few studies have investigated whether IG can suppress NOX-dependent ROS production in DSS-induced colitis. Our results confirmed experimental colon injury significantly induced the high expression of 8-OHdG, NOX1, and NOX2 compared to the normal control group. We also found IG was able to inhibit the upregulation of OHdG, NOX1, and NOX2 in a dose-dependent manner.

Recent studies have demonstrated increased production of proinflammatory cytokines including TNF-*α*, IL-2, IL-4, IL-5, IL-12, and IL-13 in IBD that are known to play a key role in the modulation of the intestinal immune system [3, 15]. Our previous studies suggested that IG could obviously reduce the levels of proinflammatory cytokines such as TNF-*α*, IL-6, and IL-8 [3, 16]. To further understand the protective roles of IG in DSS-induced colonic injury, we investigated the effects of IG on inflammation-related cytokines and the levels of IL-2, IL-4, IL-5, IL-12p40, and IL-13. In this study, DSS treatment dramatically enhanced the high expression of IL-2, IL-4, IL-5, IL-12p40, and IL-13. In our *in vitro* study, administration of IG significantly attenuated the expression of proinflammatory cytokines such as IL-2, IL-4, IL-5, IL-12p40, and IL-13 in DSS-treated rats in a concentration-dependent manner at the dose of 20-80 mg/kg. Hence, these data strongly indicated that IG could modulate the inflammatory process by inhibition of multiple inflammation-associated cytokines in experimental colitis.

Growing evidence suggests that the TLRs/MyD88/NF-*κ*B signaling pathway plays a critical role in the pathogenesis of UC and modulates the production of proinflammatory cytokines, reactive oxygen mediators, and apoptosis in the inflammatory cascade [7, 20]. TLRs recruit the signaling regulators MyD88 and TIR-domain-containing adaptor-protein-inducing interferon-*β* (TRIF) for linking to signal transduction factors via NF-*κ*B. This process further strengthens the generation of multiple proinflammatory cytokines (i.e., IL-1*β*, IL-6, and TNF-*α*) [7]. Therefore, we make further investigation to whether TLR2/4/MyD88/NF-*κ*B signaling pathways were due in part in anti-inflammation mediated by IG. In the current study, we found that IG exhibited an anti-inflammatory activity by suppressing both mRNA and protein levels of TLR2, TLR4, MyD88, and NF-*κ*Bp65 in a concentration-dependent manner. Together, these data supported the fact that IG may improve inflammation in DSS-induced colitis through blocking the TLR2/4/MyD88/NF-*κ*B signaling pathway.

## 5. Conclusions

In conclusion, we demonstrated that IG treatment significantly alleviated pathological changes in DSS-induced colitis. The evaluation of the potential mechanisms suggested that the anti-inflammation effect of IG was associated with inhibition of oxidative stress and apoptosis of intestinal epithelial cell. In addition, IG significantly suppressed DSS-induced accumulation of inflammation-associated cytokines and blocked the activation of the TLR2/4/MyD88/NF-*κ*B signaling pathway. These results indicated that IG might be a potential natural anti-inflammatory drug for the treatment of IBD. The other molecular mechanisms of these changes warranted further investigations.

## Figures and Tables

**Table 1:** Primers for real-time PCR.

mRNA species		Oligonucleotides (5′⟶3′)	Product size (bp)
TLR2	Forward	CTGTGGTATCTGAGAATGATGTGGG	239
	Reverse	TCGATGGAATCAATGATGTTGTCAA	
TLR4	Forward	CAAGACTATCATCAGTGTATCGGTGG	224
	Reverse	GCTCGTTTCTCACCCAGTCCTC	
MyD88	Forward	TTTCGACGCCTTCATCTGCTACTGC	184
	Reverse	CACCACCATGCGACGACACCTT	
NF-*κ*Bp65	Forward	CGATCTGTTTCCCCTCATCT	398
	Reverse	ATTGGGTGCGTCTTAGTGGT	
GAPDH	Forward	TTCCTACCCCCAATGTATCCG	270
	Reverse	CCACCCTGTTGCTGTAGCCATA	

## Data Availability

The data used to support the findings of this study are available from the corresponding author upon request.

## Abbreviations

IBD: Inflammatory bowel disease
UC: Ulcerative colitis
ROS: Reactive oxygen species
DSS: Dextran sulfate sodium
IG: Iridoid glycosides
DAI: Disease activity index
NOX: NADPH oxidase
8-OHdG: 8-Hydroxydeoxyguanosine
TUNEL: Terminal deoxynucleotidyl transferase- (TdT-) mediated dUTP-biotin nick end labelling
TLRs: Toll-like receptors
PCR: Polymerase chain reaction.
